# Water mold infection but not paternity induces selective filial cannibalism in a goby

**DOI:** 10.1002/ece3.2403

**Published:** 2016-09-20

**Authors:** Martin Vallon, Nils Anthes, Katja U. Heubel

**Affiliations:** ^1^ Animal Evolutionary Ecology University of Tübingen Tübingen Germany; ^2^ Present address: Institute for Zoology Grietherbusch Ecological Research Station University of Cologne Cologne Germany

**Keywords:** infanticide, kin discrimination, parental care, reproductive value, selective filial cannibalism, water mold infections

## Abstract

Many animals heavily invest in parental care but still reject at least some of their offspring. Although seemingly paradoxical, selection can favor parents to neglect offspring of particularly low reproductive value, for example, because of small survival chances. We here assess whether filial cannibalism (FC), where parents routinely eat some of their own young, is selective in response to individual offspring reproductive value. We performed two independent laboratory experiments in the common goby (*Pomatoschistus microps*) to test whether caring fathers preferentially cannibalize eggs of a given infection history and paternity. While males did not discriminate kin from nonkin eggs, they consumed significantly more eggs previously exposed to water mold compared to uninfected eggs. Our findings clearly show that parents differentiate between eggs based on differences in egg condition, and thus complement the prevailing view that FC arises for energetic reasons. By preventing the spread of microbial infections, the removal of molded eggs can constitute an important component of parental care and may represent a key driver of selective FC in a wide array of parental fish.

## 1 Introduction

Many animals invest much time and effort into the well‐being of their offspring through parental care. Yet, some of these regularly eat their very own young. This filial cannibalism (FC) appears paradoxical but is widespread across a diverse range of taxa, particularly in arthropods (Anthony, 2003; Miller & Zink, 2012; Thomas & Manica, 2003) and fish (Manica, 2002). Although FC potentially imposes direct fitness costs, it has been recognized as a reproductive strategy by which the cannibal can ultimately increase lifetime reproductive success. For instance, parents may trade‐off the survival of some offspring against their own foraging needs and use the energy gained through cannibalism to ensure continued care for the remaining current or future offspring (Rohwer, 1978; Sargent, 1992). Even cases where parents consume all of their current progeny (total filial cannibalism [TFC]) potentially increase overall fitness by enhancing future reproduction (Sargent, 1992). While such energy‐based explanations for FC dominate the literature, empirical evaluations often failed to find clear effects of energetic needs on cannibalism levels (e.g., Belles‐Isles & Fitzgerald, 1991; Klug & St Mary, 2005; Lindström & Sargent, 1997).

We here pursue the alternative idea that FC serves to discard offspring that, “for example due to sickness”, have reduced survival chances and thus low reproductive value. In fish with male brood care, where FC is particularly common (Manica, 2002), eggs often catch microbial infections. Common pathogens include water molds (oomycetes) of the genus *Saprolegnia*, which infect adults and eggs alike and pose a serious threat to egg viability (Hussein & Hatai, 2002; Kitancharoen, Hatai, & Yamamoto, 1997; Knouft, Page, & Plewa, 2003; Scott & O'Bier, 1962; van West, 2006). In the absence of brood care, eggs often rapidly overgrow with water mold hyphae and die quickly (Bronstein, 1982; Brown & Clotfelter, 2012). The caring male may actively prevent infections, for example, by secreting a protective mucus (Giacomello, Marri, Marchini, Mazzoldi, & Rasotto, 2008; Knouft et al., 2003) or by creating a constant water flow via egg fanning (Côté & Gross, 1993; St Mary, Gordon, & Hale, 2004). However, when these measures fail, selective removal of sick offspring through FC might provide an efficient ultimate treatment against infection threats with the added benefit of yielding some energy via egg consumption. Although briefly discussed before (Hoelzer, 1988), this possibility has to date largely received anecdotal support (Bailey, 1952; Kraak, 1996; Winn, 1958), with the exception of a very recent study that demonstrated targeted removal of eggs carrying natural *Saprolegnia* infections in spottail darters, *Etheostoma squamiceps* (Bandoli, 2016). Our study complements this work by providing the first rigorous test for selective FC after experimental manipulation of water mold infection.

Besides dead or sick offspring, many animal fathers face the risk of caring for offspring sired by other males. A particularly high uncertainty of paternity occurs in many bird (Griffith, Owens, & Thuman, 2002) and fish species (Taborsky, 1994). In fishes, so‐called sneaker males may sneak fertilizations of eggs at another male's nest, leaving the guarding male with foreign (nonkin) eggs to care for (Taborsky, 1994). Selection should favor males that recognize and cannibalize such foreign offspring and thereby not only avoid allocating costly paternal care to unrelated eggs but also use those as a cheap energy source. Recent evidence indicates that selective cannibalism of foreign eggs indeed occurs in some (Green, Mirza, & Pyle, 2008; Mehlis, Bakker, Engqvist, & Frommen, 2010; Neff, 2003a) but clearly not all fish (Bandoli, 2006; DeWoody, Fletcher, Wilkins, & Avise, 2001; Lissåker & Svensson, 2008), calling for further studies to evaluate its prevalence.

**Figure 1:**
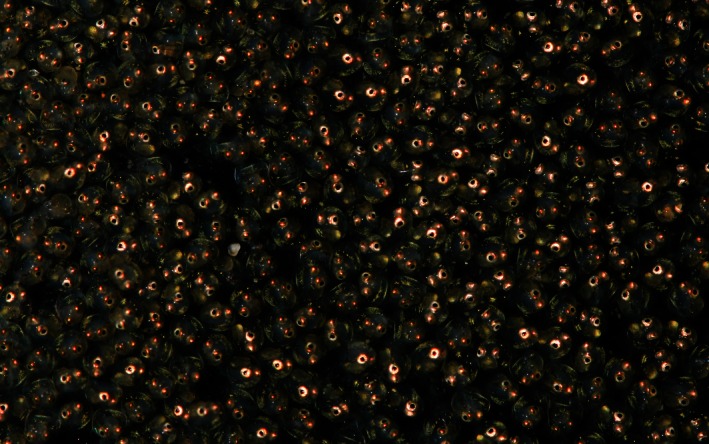
*Pomatoschistus microps* eggs close to hatching with embryos already clearly visible

In this study, we investigated the influence of water mold infections and kinship on FC in a small marine fish with exclusive male care and common sneaking behavior (Magnhagen, 1992), the common goby (*Pomatoschistus microps*, Krøyer, Fig. 1), where recent circumstantial evidence indicated that kinship has no effect on FC (Vallon & Heubel, 2016). Nest‐holding males received infected and uninfected eggs simultaneously, which were either sired by themselves (“own egg” group) or an unrelated male (“foreign egg” group). To obtain a robust assessment of cannibalism patterns, we conducted two independent replicate experiments following slightly different methodological approaches.

## 2 Material and Methods

### 2.1 Model system

Reproduction in common gobies is restricted to a single breeding season (from May to August), but individuals can complete several consecutive breeding cycles during this period (Miller, 1975). Males build nests using mussel shells (or rarely other solid objects) and sand, and competition for such nest sites can be fierce (Borg, Forsgren, & Magnhagen, 2002; Nyman, 1953). Nest‐holding males try to attract females and can potentially acquire clutches from several females if the nest is large enough. The deposition of eggs by a female can take several hours (Nyman, 1953), and smaller sneaker males may try to enter the nest and sneak fertilizations of unfertilized eggs (Magnhagen, 1992; Svensson, Magnhagen, Forsgren, & Kvarnemo, 1998). The nest‐holding male cares for the eggs until hatching (for about 5–12 days, depending on temperature; MV, unpublished data) while the female abandons the nest immediately after spawning. Paternal care includes fending off predators, maintaining the nest, and cleaning and ventilating the eggs (Nyman, 1953), but males also show frequent FC (Kvarnemo, Svensson, & Forsgren, 1998; Svensson et al., 1998).

### 2.2 General setup

**Figure 2:**
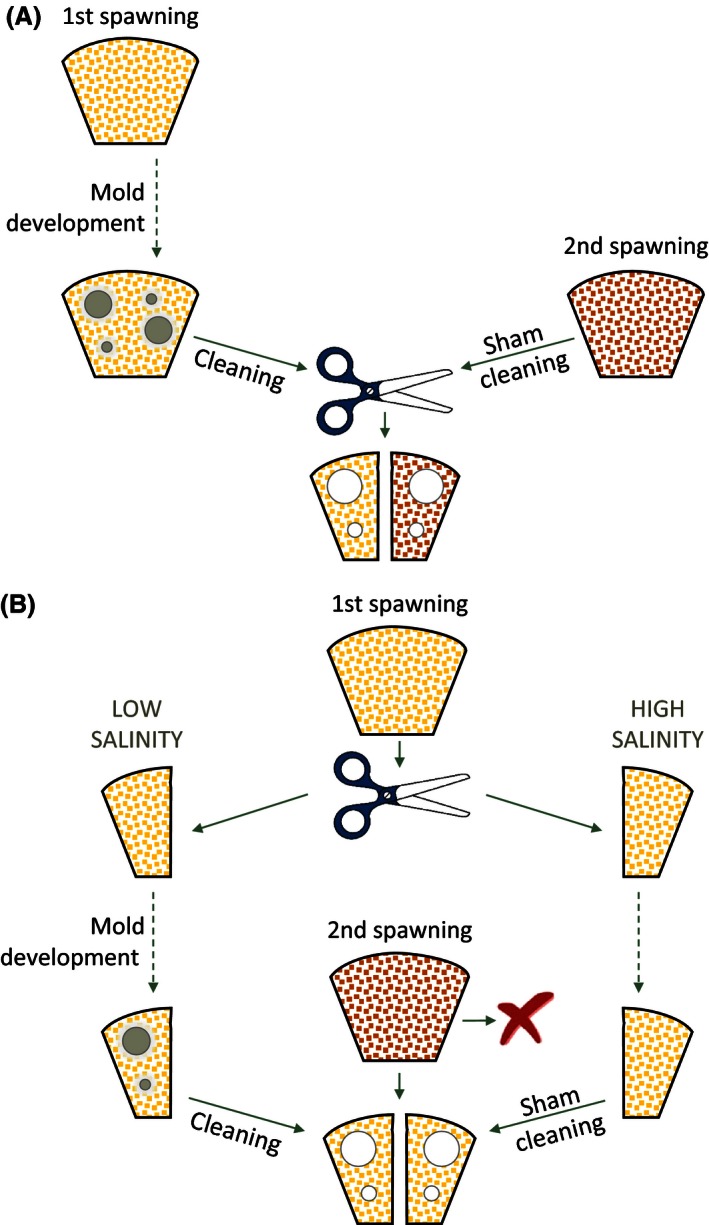
Schematic overview over experimental procedures in (A) experiment 1 and (B) experiment 2. Note that in both experiments, there was a second group of males that received foreign instead of their own eggs after the water mold treatment. See main text for details

To test how FC is affected by egg infection and kinship (i.e., caring for own versus foreign eggs), we conducted two separate experiments that followed a similar general setup and mainly differed in the approach used to induce the growth of water mold on the eggs (Fig. 2). The experiments were conducted at Tvärminne Zoological Station in southern Finland in June 2013 (2.3.1) and June and July 2014 (2.3.2). Male and female common gobies were caught nearby, close to the shoreline at Henriksberg (59°49.75′N, 23°08.67′E) using a seine. Additional males were collected in the same bay from previously installed artificial nests (ceramic tiles) using hand nets. We measured body size of all fish as total length (TL) to the nearest mm. Males were housed individually in 35 L test aquaria and given 3 days to acclimatize to the aquarium environment while females were kept in stock tanks prior to use. To avoid interactions between neighboring males, all test aquaria were covered with black foil. Each test aquarium was equipped with a halved flowerpot (4.5 cm diameter) placed upside‐down, which served as an artificial nest site and was fitted with a removable plastic sheet for females to spawn on. A flow‐through system continuously supplied all aquaria with fresh seawater. Water temperature thus mirrored natural conditions and was measured daily. All fish experienced a standardized light regime (18 L:6 D), and individual males were fed with two frozen chironomid larvae twice daily.

### 2.3 Treatments and procedures

#### 2.3.1 Experiment 1

In this experiment, each male consecutively spawned once with each of two different females. The first clutch was exposed to water mold as detailed below, and the second one maintained without water mold. The two clutches per male were halved and recombined into two clutches, each containing 50% molded old and 50% unmolded new eggs (Fig. 2A). One of these clutches was then returned to the original father (“own eggs” group), the other to an unrelated (nonkin) male (“foreign eggs” group). As a result, this experiment allowed us to simultaneously assess the effect of water mold infection and kinship on FC.

Short‐term removal of egg masses is a well‐established method in goby ecology with no handling effects on egg survival or parental care (Jones & Reynolds, 1999; Vallon & Heubel, 2016; Vallon et al., 2016).

However, note that the water mold treatment in this experiment confounds with egg age such that molded clutch halves contain older eggs. This confounding is conservative, however, because goby males generally value older eggs clearly higher than younger eggs (Klug & Lindström, 2008; Vallon & Heubel, 2016, see discussion). Moreover, experiment 2 (as detailed below) uses a modified experimental paradigm that excludes this confounding while yielding qualitatively similar results.

For spawning, each of initially 48 males was exposed to one female (mean ± *SE* TL: 35.7 ± 0.4 mm) for 16–18 h overnight. Males that had not received a clutch of eggs were paired with a new female up to three more times and excluded if still unsuccessful. The second spawning was initiated 3 days later with an identical approach (mean ± *SE* female TL: 36.3 ± 0.6 mm, mean ± *SE* time difference between first and second clutch: 4.0 ± 0.2 days). Twenty‐six males did not spawn with two successive females and were thus excluded from further analysis.

All males that completed their two consecutive spawnings were then assigned to the “own egg” group (mean ± *SE* TL: 34.0 ± 0.6 mm, *n* = 13) or the “foreign egg” group (34.3 ± 0.9 mm, *n* = 9). All egg masses acquired by males assigned to the “foreign egg” group were now discarded—these spawnings exclusively served to initiate paternal care behavior now dedicated to their newly allocated “foreign eggs.” First clutches of males in the “own egg” group were individually labeled, photographed for subsequent determination of clutch size, and placed in one of two 73 L aerated rearing tanks (up to 20 clutches per tank) without a male (Fig. 2A). To simulate nest conditions, rearing tanks were kept under low light and the plastic sheets with eggs were pinned upside‐down to styrofoam plates floating at the surface. Due to the lack of paternal care, egg mold was gradually developing on all these clutches. Individual clutches were removed from the rearing tanks as soon as the corresponding male had acquired a second clutch. To reduce the risk that males rejected entire clutches just due to untypically progressed water mold infection, we removed all eggs visibly overgrown with water mold hyphae by scraping them off the plastic sheet using scissors and retaining only eggs that visually appeared healthy but were previously exposed to water mold, and thus likely still carried the infection. A similar number of eggs were removed from the second mold‐free clutch as a sham treatment. Both clutches of a given male were cut in halves and recombined into two experimental clutches, each containing a molded and an unmolded half fertilized by the same sire. The new mixed clutches contained similar numbers of healthy eggs (mean ± *SE* number of eggs: 292.4 ± 32.5) and eggs exposed to water mold (314.0 ± 30.5 eggs; paired *t* test; *t *=* *−1.24, *df* = 21, *p *=* *.228). One experimental clutch each was now placed into the father's (“own egg” group) and the foreign male's (“foreign egg” group) nests, with two plastic clips holding the egg sheets in place. All males resumed paternal care on egg insertion.

#### 2.3.2 Experiment 2

The overall experimental paradigm closely resembled that of experiment 1, with the exception that experiment 2 manipulated water mold infection levels independent of age within single clutches (Fig. 2B) as detailed below.

Males assigned to either the “own egg” or the “foreign egg” kinship groups (mean ± *SE* TL of males used in the main analysis; own eggs: 34.1 ± 0.5 mm, *n* = 22; foreign eggs: 35.3 ± 0.6 mm, *n* = 16; *t* test; *t *=* *−1.61, *df* = 36, *p *=* *.116) again spawned twice as outlined for experiment 1 with two similar‐sized females (mean ± *SE* TL; female 1: 34.9 ± 0.3 mm; female 2: 35.1 ± 0.4 mm), now with just 2 days between spawnings (31 of initially 73 males did not spawn twice and could not be used). However, only the first clutches of “own egg” males were kept for the experiment and immediately split into two similar‐sized halves that were exposed to two different salinities (mean ± *SE* number of eggs; low salinity: 403.7 ± 16.1; high salinity: 410.6 ± 15.9; Fig. 2B). As in experiment 1, own clutches of males assigned to the “foreign egg” group were only required to initiate paternal care behavior and thus discarded. Instead, the “foreign egg” males now obtained their fostered eggs from a third group of males (*n* = 16) that spawned just once, with these clutches halved and exposed to salinity treatments as outlined above for “own egg” males.

The salinity treatment draws from the well‐known effects of salinity on water molds such as *Saprolegnia* (Ali, 2005; Marking, Rach, & Schreier, 1994) to establish two groups with clear differences in water mold infection risk. The low salinity treatment (mean ± *SE*: 6.36 ± 0.02 ppt) allowed water mold growth (see 2.3.1) under conditions mimicking salinity at the study site (typically between 6.2 and 6.4 ppt). The high salinity treatment (18.52 ± 0.05 ppt) mimicked conditions under which the closely related sand goby (*Pomatoschistus minutus*) showed drastically reduced water mold growth, and hence lower infection risk (Lehtonen & Kvarnemo, 2015a). Salinity differences only applied to the artificial rearing period to manipulate water mold infections. All mating, spawning, and fertilization as well as paternal care and FC took place under natural ambient salinity conditions identical to those in experiment 1. Thus, there was no direct link between salinity and fish behavior.

Salinity treatments were created by mixing 50% purified water (Milli‐Q) with 50% natural Baltic Sea water and adding the appropriate amount of a sea salt mix for marine aquaria (Instant Ocean, Aquarium Systems, Sarrebourg, France). The rearing tank setup was similar to experiment 1, but we used small plastic tanks (6 L) and limited the number of clutches per tank to four to minimize the risk of infections spreading to neighboring clutches. Furthermore, we refrained from using a flow‐through system for the rearing tanks to be able to maintain stable salinity levels. We instead manually exchanged approximately 50% of the water volume in each tank every second to third day. Tanks with different salinities were spatially alternated and the two differently treated clutch halves per male were always positioned in directly neighboring tanks. Average water temperature was nearly identical in both treatments (mean ± *SE*; low salinity: 14.51 ± 0.10°C; high salinity: 14.52 ± 0.10°C). We visually inspected the health status of the eggs once per day.

**Figure 3:**
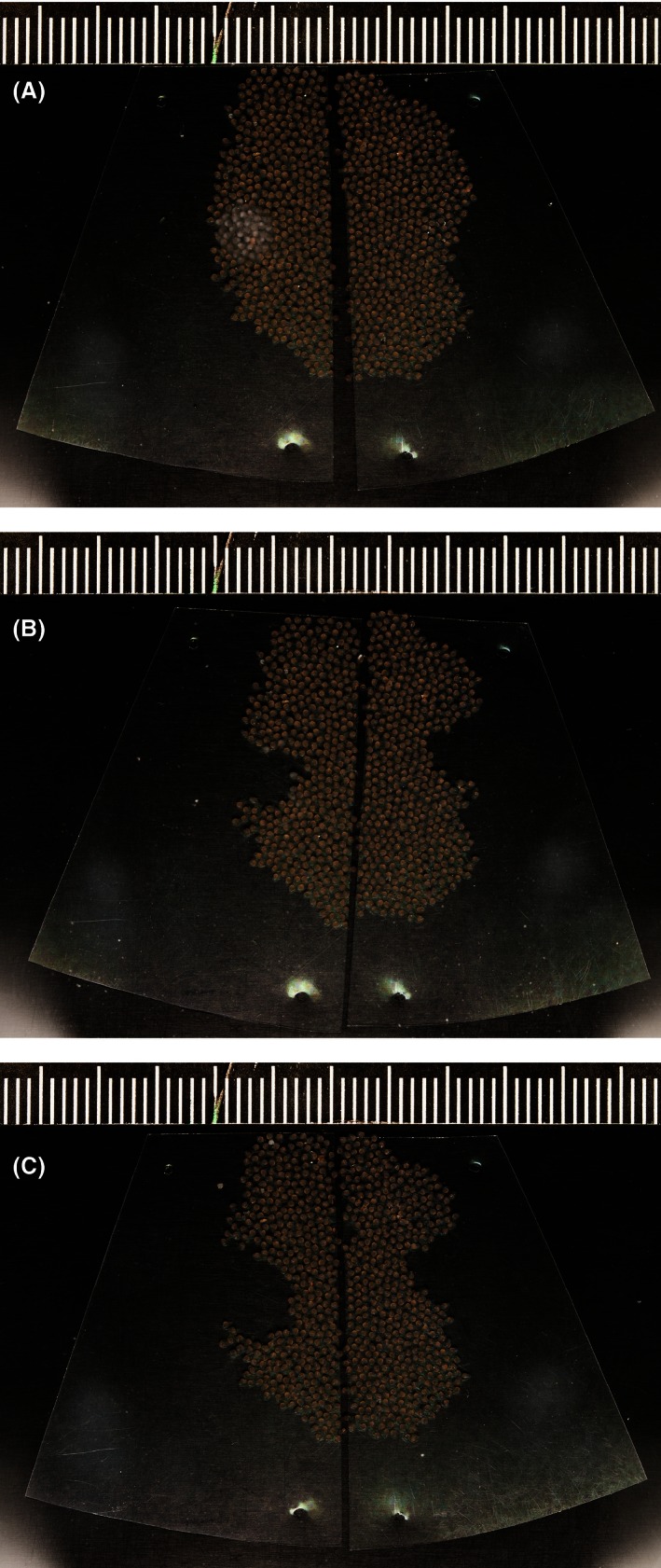
Consecutive sample photographs of a single clutch from experiment 2, showing the clutch after (A) salinity treatment, (B) cleaning and sham cleaning, and (C) 1 day with the male. Note water mold growth on the left half in (A) and visible filial cannibalism in (C)

**Figure 4:**
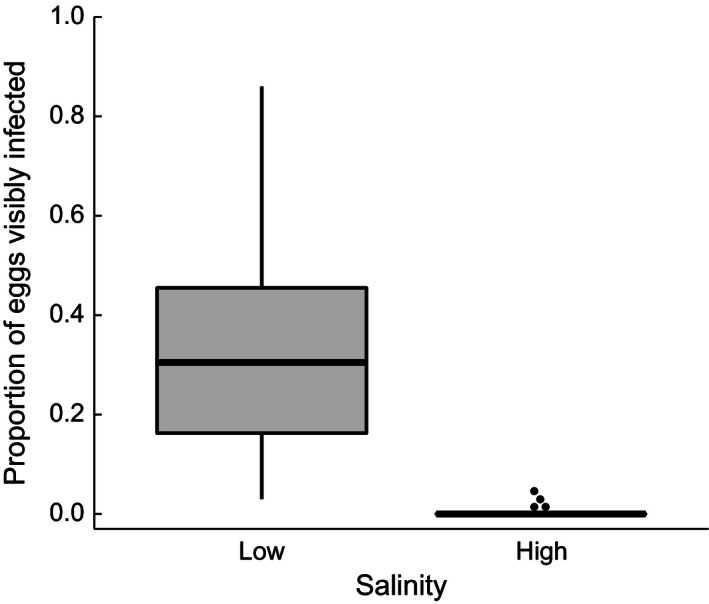
Proportion of eggs visibly infected with water mold on the differently treated clutch halves (*n* = 42 per treatment) in experiment 2

The experimental phase was initiated when a given male acquired its second clutch (which was exclusively needed to trigger paternal care behavior) and when we could confirm sufficient water mold growth on the low salinity half of the treated clutch halves from the first spawning (mean ± *SE* duration of artificial rearing: 3.6 ± 0.2 days). The two clutch halves were removed from their rearing tanks and photographed (Fig. 3A) to accurately determine whether the salinity treatment was successful. There was indeed a drastic difference in the proportion of eggs overgrown by water mold with virtually no water mold growing on the high salinity halves (Fig. 4). To maximize the difference between treatments, we excluded four males with visible water mold growth under high salinity (see outliers in Fig. 4). Due to this clear distinction, we hereafter refer to the low salinity half as the “molded” half and to the high salinity one as the “unmolded” half.

Similar to experiment 1, we scraped off the visibly infected parts on the molded half and removed a similar pattern of eggs as a sham treatment from the unmolded half. We then took another picture of both halves combined as a baseline for FC measurements (Fig. 3B). The two halves did not differ in egg number (mean ± *SE* eggs; unmolded: 289.5 ± 20.5; molded: 298.5 ± 19.6; paired *t* test; *t *=* −*1.35, *df* = 37, *p *=* *.184). Finally, we swapped the combined clutch with the clutch the male (either “own eggs” or “foreign eggs” group) was currently caring for and discarded this second clutch.

### 2.4 Data collection and statistical analysis

Data collection and analysis were performed in a similar manner in both experiments. One day after a male had received the treated clutch halves, we removed and photographed them (Fig. 3C). The male was then released into the wild. To quantify original clutch size and FC, we counted eggs manually on all consecutive images using the Cell Counter plugin (Kurt De Vos, University of Sheffield, UK) in ImageJ version 1.47v (Wayne Rasband, NIH, USA). We obtained FC measurements for 22 males in experiment 1 and 38 males in experiment 2. However, in each experiment, one male consumed the entire mixed clutch. Given that TFC is typically seen as a distinct behavior examined separately from partial clutch consumption (Manica, 2002; Sargent, 1992), we excluded these males from the main analysis.

To statistically compare cannibalism levels between molded and unmolded eggs and between “own egg” and “foreign egg” males, we fitted generalized linear‐mixed models (GLMMs) with binomial error distributions using the “lme4” package version 1.1‐6 (Bates, Maechler, Bolker, & Walker, 2015) in R v. 3.0.3 (R Core Team, 2014). Our response variable derived the proportion of cannibalized eggs directly from the number eggs eaten versus those remaining untouched, and thus incorporated a measure of initial egg number (see Vallon & Heubel, 2016 and references therein). We included *Male ID* as a random factor with random intercepts and slopes over water mold treatments to reflect that each male provided paired data for cannibalism on molded and unmolded clutch halves. Fixed factors were *mold* (no or yes; i.e., unmolded or molded), *kinship* (own eggs or foreign eggs), and their interaction. In addition, we added *male length* as a covariate, which was *z*‐transformed to improve model convergence (Korner‐Nievergelt et al., 2015). Both models were reduced to the most parsimonious ones using the Bayesian information criterion (Zuur, Ieno, Walker, Saveliev, & Smith, 2009) and ultimately contained only the fixed factors *mold* and *kinship* (retained as one of the main treatment factors) as neither their interaction nor the covariate *male length* improved model fit.

For experiment 2, we additionally tested in a separate binomial GLMM if FC was related to the proportion of infected eggs initially present on the low salinity half (corresponding data not available for experiment 1), while correcting for overdispersion in this model by including an observation‐level random factor (Gelman & Hill, 2007; Korner‐Nievergelt et al., 2015).

## 3 Results

**Figure 5:**
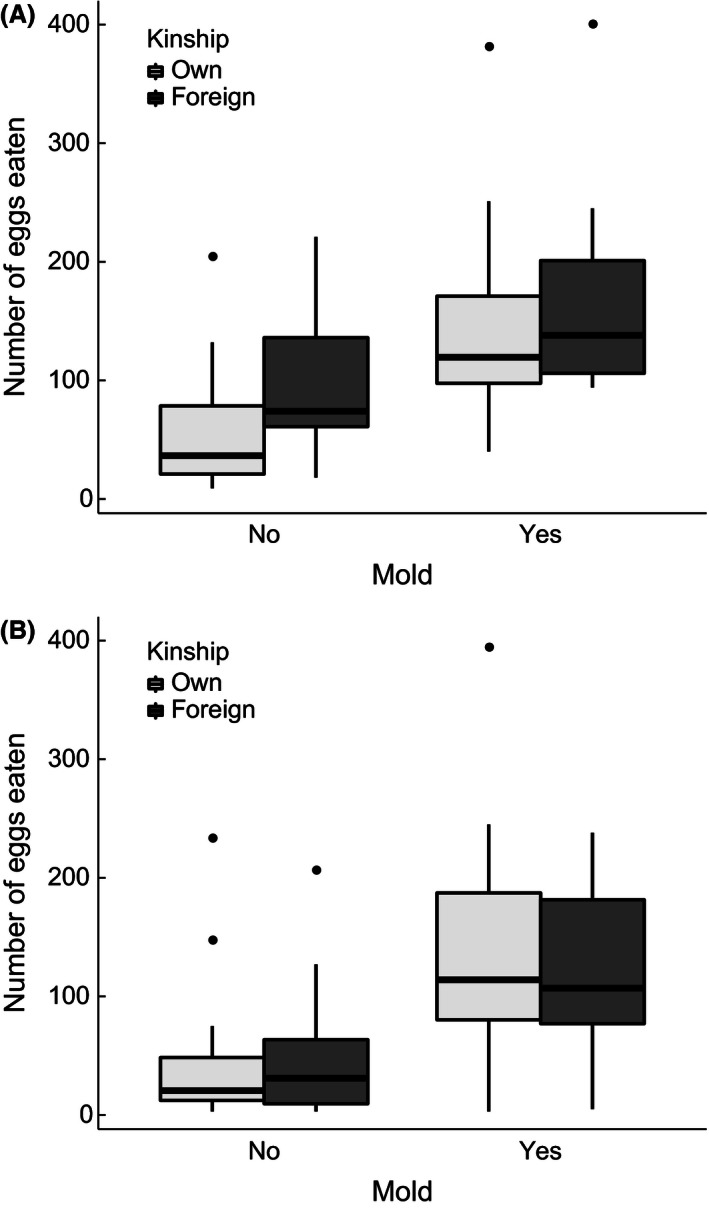
Filial cannibalism after 1 day in (A) experiment 1 and (B) experiment 2. The graphs show raw numbers of cannibalized eggs, and thus complement the proportions given in the main text and the underlying statistical analysis. See main text for sample sizes

In agreement with our main prediction, we found that in experiment 1, males cannibalized a significantly higher proportion of eggs from the molded half (mean ± *SE*: 56.4% ± 5.7%) than from the unmolded half (34.3% ± 6.4%; *n* = 42 observations of 21 individuals, *z* = 4.92, *p *<* *.0001; Fig. 5A). In contrast, average cannibalism did not significantly differ between males caring for own (40.8% ± 6.5%) and males caring for foreign eggs (51.4% ± 6.2%; *n*_own_ = 12, *n*_foreign_ = 9, *z* = 0.87, *p *=* *.383; Fig. 5A).

**Figure 6:**
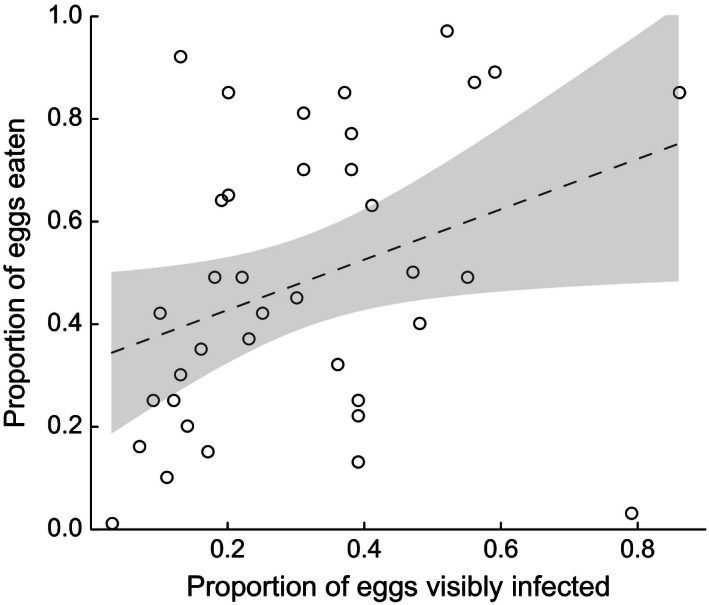
Proportion of eggs cannibalized after 1 day on the molded halves (low salinity treatment) in experiment 2 in relation to the proportion of molded eggs initially visible (*n* = 37). The gray area depicts the 95% confidence interval of the regression line

The pattern was similar in experiment 2. We found a strong positive effect on cannibalism of water mold infection (48.2% ± 4.6% of the eggs from the molded half compared to 16.6% ± 3.1% from the unmolded half; *n* = 74 observations of 37 individuals, *z* = 12.70, *p *<* *.0001; Fig. 5B) but none of kinship (31.5% ± 4.2% of own compared to 33.9% ± 5.5% of foreign eggs; *n*_own_ = 22, *n*_foreign_ = 15, *z* = 0.38, *p *=* *.705; Fig. 5B). In addition, within the molded clutch halves, FC increased with the proportion of visibly molded eggs present before the cleaning procedure (*n* = 37, *z* = 2.06, *p *<* *.0392; Fig. 6).

## 4 Discussion

Using two independent experiments with common gobies as a model system, we show that water mold infection of eggs strongly affects FC. Caring males preferentially consumed eggs that had been exposed to a water mold environment. Our results thus clearly support the hypothesis that FC, at least partly, serves the purpose of removing diseased offspring. By doing so, the cannibal can clear the nest of eggs which will likely never hatch, and thus essentially have no reproductive value, while using the infected eggs as an additional food source. The energy gained by egg consumption could in turn facilitate taking care of the remaining eggs (Rohwer, 1978; Sargent, 1992). In consequence, such selective FC may be a mechanism to re‐allocate paternal care to offspring of higher reproductive value (Klug, Alonzo, & Bonsall, 2012).

In the context of water mold infections, egg cannibalism likely also serves to preventing the spread of the disease. Two recent laboratory studies assessed the effect of egg density (either via natural variation or manipulation) and salinity on egg viability using artificially reared sand goby clutches (Lehtonen & Kvarnemo, 2015a,c). They found that under low but not under high salinity conditions (where water mold growth is inhibited), clutches with low egg densities had a lower prevalence of *Saprolegnia* and increased egg survival compared to clutches with high egg densities, indicating that low egg densities lead to a reduced spread of infections. Correspondingly, there is a clear evidence from rainbow trout (*Oncorhynchus mykiss*) and Atlantic salmon (*Salmo salar*) that *Saprolegnia* infections on egg clutches spread to healthy eggs mainly by hyphal growth from adjacent infected eggs rather than (randomly) by zoospores (Smith, Armstrong, Springate, & Barker, 1985; Thoen, Evensen, & Skaar, 2011). This suggests that removing damaged or already dead eggs reduces the risk that surrounding eggs get infected.

Interestingly, we found in experiment 2 that male cannibalism on molded clutch halves increased with the proportion of eggs that was visibly molded after water mold exposure, even though these visibly molded eggs were experimentally removed before returning the clutch to the male (Fig. 6). This indicates that when many visibly moldy eggs had been present, there were also many potentially infected adjacent eggs, leaving more targets for selective FC. In agreement, we observed that males mostly cannibalized eggs directly neighboring the previously molded, and thus removed clutch parts (Fig. 3), while cannibalism occurred spatially more randomly on the sham‐treated, unmolded clutch halves (MV, personal observation). In some cases, individual eggs that we identified as seemingly unhealthy during image analysis (e.g., because they became opaque) were missing on subsequent pictures, indicating that males specifically pick out single eggs. How fish recognize infected eggs cannot be answered with our design and provides a highly interesting research question for future studies. Recent evidence from sand gobies indicates a possible role for olfactory cues, because females avoided to lay eggs into nests that “smelled” of water mold when given the choice between nests either with or without a *Saprolegnia* odor (Lehtonen & Kvarnemo, 2015b).

In experiment 1, we prevented paternal care in one treatment for approx. 4 days to induce water mold growth on eggs while using newly laid eggs without water mold as comparison, thus additionally introducing a difference in egg age between treatments. Nevertheless, we are confident that our results are primarily caused by the difference in water mold growth. In a previous study, we found that males preferentially consume younger eggs when given the choice, presumably due to their lower reproductive value compared to more developed eggs (Vallon & Heubel, 2016). In contrast, males cannibalized the theoretically more valuable (but infected) older eggs in the present study, indicating that water mold infections override the benefits of favoring more developed eggs. In experiment 2, the presence of egg mold was manipulated via salinity and independent of egg age. We cannot entirely rule out that higher salinity had effects on egg development beyond inhibiting water mold growth, but are confident that confounding should be mild at maximum for various reasons. First, previous evidence suggests that common goby eggs (even of marine origin) develop similarly well in 6 and 18 ppt salinities at the temperature used in our experiment (Fonds & Van Buurt, 1974), suggesting a short rearing period in manipulated salinities should not adversely affect egg development in either treatment. Second, both egg laying and paternal care for these eggs all occurred in a common environment, excluding direct physiological or behavioral parental effects on egg condition triggered by ambient water. Third, we expect the fish and eggs in our experiment to be more adapted to the low salinity conditions because the chosen treatment closely mimicked the prevailing conditions of the study population (see 2). Assuming at least some degree of local adaptation, our finding that eggs raised under these conditions were preferentially cannibalized over those exposed to a non‐native, high salinity is rather opposite to the expected confounding effects. Finally, we considered manipulating water mold infections indirectly through salinity superior to direct infection in that the expressed water mold infections reflects an ecologically relevant degree of variation. Taken together, our two complementary experiments clearly highlight the importance of water mold infections for selective FC.

In contrast to water mold infections, kinship did not affect the amount of partial FC shown by males in both experiments. In addition, while we would expect nest owners to eat all and not just a fraction of the foreign eggs if they were capable to discriminate against eggs fertilized by a different father, total FC was near‐absent in all our experiments. These results confirm indications from a previous study in common gobies (Vallon & Heubel, 2016), also finding no difference in FC between males caring for their own eggs and so‐called surrogate males caring for foreign eggs. However, this finding was not as clear as in the present study due to methodological limitations leading to confounding between the two kinship groups. In general, the evidence for selective FC of foreign eggs is mixed (see references in the 1) and this behavior could well be species specific. Discriminating between own and foreign offspring may be difficult to start with. In fish, olfactory cues have been suggested to be more important than visual cues (Frommen, Brendler, & Bakker, 2007; Loiselle, 1983; Mehlis et al., 2010). We measured FC on average 4.8 days after egg deposition in our study (excluding the second clutches of experiment 1, which were only 1 day old). Possibly, paternal odor cues are only sufficiently present at later stages in development (discussed in Mehlis et al., 2010). For instance, bluegill sunfish (*Lepomis macrochirus*) are only able to recognize foreign offspring after the eggs have hatched, probably due to previously absent urinary cues (Neff, 2003b). However, for FC in common gobies, kin recognition after hatching is probably meaningless because FC is relevant only during the egg caring period and larvae leave the nest soon after hatching and live independently (Nyman, 1953).

Males may alternatively not directly discriminate own from foreign offspring, but rather use external cues to infer the risk of paternity losses, and thus to assess the value of their brood. Males of several fish species decrease paternal effort (Neff, 2003b) or increase FC (Gray, Dill, & McKinnon, 2007; Manica, 2004) when potential sneaker males are present during spawning. Such an indirect mechanism would not have been detectable in our setup. However, corresponding studies in *Pomatoschistus* gobies did not detect an effect of sneaker presence on FC (common goby: Svensson et al., 1998; sand goby: Svensson & Kvarnemo, 2007), although nest‐holder males generally react strongly to sneaker males in both species (Magnhagen, 1998; Malavasi, Lindström, & Sundström, 2001; Svensson & Kvarnemo, 2007). Interestingly, genetic data from experimental studies in sand gobies suggest that the paternity of nest‐holding males remains high even after successful sneaking (Malavasi et al., 2001; Svensson & Kvarnemo, 2007). Assuming a similar pattern for common gobies, one may argue that direct or indirect mechanisms for offspring recognition never developed in these species because of just marginal costs of caring for a comparatively small fraction of foreign eggs.

To conclude, this study provides independently replicated experimental evidence that selective FC strongly responds to water mold infection but not to kinship. The common occurrence of microbial egg infections in fish on the one hand and FC on the other hand suggests that the documented link between both phenomena may not be restricted to common gobies but rather widespread at least in fish, consistent with recent findings in spottail darters (Bandoli, 2016). While FC is potentially influenced by a wide range of different (but not necessarily mutually exclusive) factors (Klug & Bonsall, 2007; Manica, 2002), our study therefore isolates removal of sick or dead offspring as one of its main drivers.

## Ethics

The study complies with all the relevant laws of Finland and was approved by Finnish authorities. All procedures were declared as class 0 experiments and inspected and approved by ELLA, Animal Experimental Board in Finland.

## Data Accessibility

All data associated with this article will be archived at the Dryad Digital Repository: http://dx.doi.org/10.5061/dryad.1gk0m.

## Funding Information

This project was financially supported by a grant from the Volkswagen foundation to KUH (grant number I/84 846).

## Conflict of Interests

We have no competing interests.
